# Short-term intratracheal use of PEG-modified IL-2 and glucocorticoid persistently alleviates asthma in a mouse model

**DOI:** 10.1038/srep31562

**Published:** 2016-08-16

**Authors:** Kefei Wu, Jiexian Ma, Weiya Bai, Xiaoxian Cui, Tao Han, Shiyuan Wang, Youhua Xie, Yanhui Xie

**Affiliations:** 1 Department of Hematology, Huadong Hospital, Shanghai Medical College, Fudan University, Shanghai 200040, People’s Republic of China; 2 Key laboratory of medical molecular virology, Institutes of biomedical sciences and institute of medical microbiology, School of Basic Medical Sciences, Shanghai Medical College, Fudan University, Shanghai 200032, People’s Republic of China; 3 Xiamen Amoytop Biotech Co., Ltd, Xiamen 360000, People’s Republic of China

## Abstract

Regulatory T (Treg) cells play an important role in allergic airway diseases, and upregulation of Treg cells is a potential therapeutic strategy for asthma. In this study, we show that short-term intratracheal use of IL-2 combined with glucocorticoid alleviates antigen-induced airway inflammation and reduces airway hyperresponsiveness by expanding antigen-nonspecific Treg cells, with a decrease in T helper 2 (Th2) cells and Th2-associated cytokines. We also designed a long-acting polyethylene glycol (PEG)-modified IL-2 and demonstrated that the optimal dosage form is IL-2(PEG) plus budesonide, which can upregulate Treg cells and ameliorate asthma at a lower dose. The therapeutic effect was faster than treatment with dexamethasone and was effective at a low dose suitable for humans that could last for at least 6 weeks. This study unveils a new therapeutic regimen and suggests that such endogenous Treg therapy could be a useful tool to persistently alleviate asthma.

The incidence of asthma, a common inflammatory airway disease that can eventually cause irreversible airway narrowing, is increasing throughout the world, imposing enormous burdens on health care systems in both developed and developing countries^1^,^2^. Characterized by abnormal activation of innate and adaptive immune cells, the pathological process of asthma involves eosinophilic pulmonary inflammation, mucus production and airway obstruction and the pathological course of disease can be prolonged. CD4+CD25+ regulatory T (Treg) cells, which play an important role in the maintenance of peripheral tolerance, have been reported to be responsible for the prevention of allergic airway diseases, and the lack of Treg cells promotes the pathological process of asthma^3^,^4^,^5^.

An infusion of Treg cells has proven to be successful in alleviating airway inflammation and hyperreactivity in mouse model. However, this method is restricted by many factors, including the complex process and the need for sophisticated equipment^6^,^7^. Pharmacological manipulations, such as steroids and IL-2/IL-2 antibodies, have successfully expanded Treg cells and alleviated asthma *in vivo* in mouse models^8^,^9^. However, the side effects resulting from long-term, high-dose administration of steroids and a restriction on the preventive use of IL-2/IL-2 antibody in humans limits the clinical application of these methods. It has been reported that short-term intraperitoneal administration of dexamethasone and IL-2 can markedly expand CD4+CD25+ FoxP3+ Treg cells and alleviate experimental autoimmune encephalomyelitis (EAE) and asthma in mouse models^10^,^11^. However, such systemic treatment can influence the general immune system, and a relatively large number of enhanced functions of Treg cells can weaken anti-tumor and anti-infection immune responses, resulting in an immunocompromised state. Furthermore, the optimal dose in systemic treatment of asthma is too high to be suitable for use in humans^11^. In addition, this invasive therapy may hinder patient compliance.

In asthma, antigen uptake in alveoli gives rise to accumulation of dendritic cells (DCs) and antigen retention in the airway-adjacent region and then stimulated specific T cells also accumulate in the airway-adjacent region after allergen challenge and are activated by the accumulated DCs^12^. This environment provides a key precondition for local drug application, in that the targeted T cells and drugs can interact with each other in the airways.

In this study, we show that local application of glucocorticoid and IL-2 via the airway can effectively upregulate Treg cells and alleviate the pathological process of asthma in mice model. We also determine the optimal dosage form of glucocorticoid and IL-2 and the best dose of the two drugs per mouse. Our study suggests that intratracheal treatment with glucocorticoid combined with IL-2 is a safe and effective pharmacological manipulation in asthma therapy in mice and could potentially be used in humans.

## Results

### Short-term intratracheal use of IL-2 combined with dexamethasone suppresses allergic airway disease

**Figure 1:**
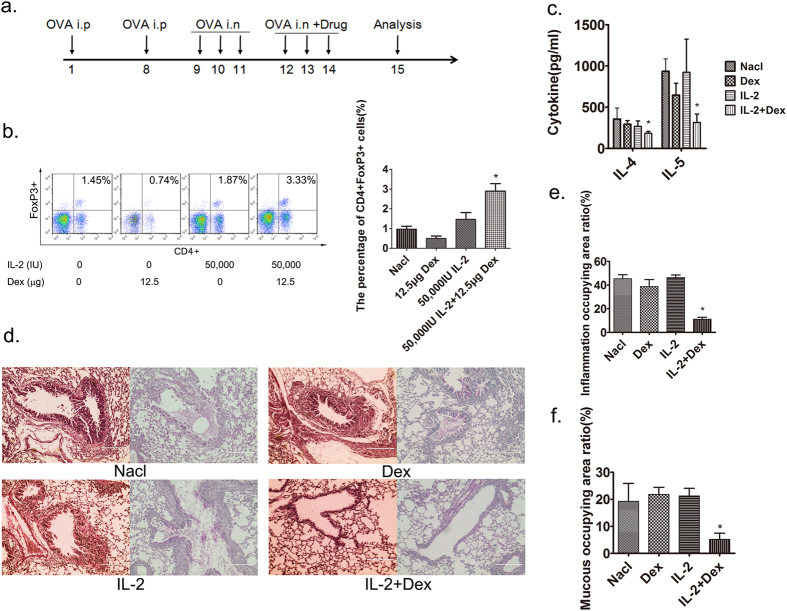
Manifestations of allergic airway disease after intratracheal use of IL-2 plus dexamethasone. Intratracheal administration of IL-2 and Dexamethasone upregulated Tregs in BALF and alleviated asthma. (**a**) Timeline of drug intervention and analysis. Female BALB/c mice were immunized with OVA i.p on days 1 and 8, followed by intranasal (i.n) 2% OVA challenges on days 9–14. And 50,000 IU IL-2 plus 12.5 μg dexamethasone (Dex) were administrated intratracheally on days 12–14. On day 15, mice were sacrificed and analyzed by flow cytometry and histopathology. (**b**) Detection of CD4+FoxP3+ Treg cell composition among lymphocytes in BALF by flow cytometry. (**c**) Analysis of Th2 cytokines IL-4 and IL-5 in BALF. (**d**) H&E and PAS staining of the lung section (scale bars, 200 μm). (**e,f**) Computer-based quantification of lung inflammation and mucus production. *p < 0.05, 50,000 IU IL-2 plus 12.5 μg dexamethasone group versus other groups by two-way ANOVA analysis. Data are presented as means ± SEM (n ≥ 8 per group and data point) from 2 independent experiments. *i.n*., Intranasal; *i.p*., intraperitoneal. Nacl group, asthma model mice treated with normal saline.

In our previous study, we demonstrated that short-term intraperitoneal use of IL-2 at a dose of 400,000 IU combined with dexamethasone at a corresponding dose of 100 μg per mouse could upregulate Treg cells effectively and alleviate allergic airway disease in an asthma mouse model^11^. In the current study, to evaluate the therapeutic effect of local administration, using ovalbumin (OVA) as an experimental allergen, asthma model mice were treated intratracheally with a fixed ratio of IL-2 and dexamethasone as reported before (50,000 IU IL-2: 12.5 μg dexamethasone versus 400,000 IU IL-2: 100 μg dexamethasone)^11^. We identified Treg cells based on expression of CD4 and FoxP3^13^. Three days of treatment with normal saline, IL-2 alone or dexamethasone alone failed to upregulate Treg cells, whereas treatment with IL-2 plus dexamethasone markedly upregulated Treg cells in bronchoalveolar lavage fluid (BALF) (Fig. 1a,b). To investigate the therapeutic effect on cytokine production, the Th2 cytokines IL-4 and IL-5 in BALF, which are involved in the pathogenesis of asthma, were analyzed and found significantly decreased (Fig. 1c). As for airway inflammation, combined use of IL-2 and dexamethasone could significantly reduced bronchial inflammation and mucus production, which were verified by investigator-independent computer-based analysis of H&E or PAS-stained lung sections. (Fig. 1d–f).

### Upregulation of Treg cells is dose-dependent and IL-2 (PEG) combined with budesonide proves to be effective at low doses

**Figure 2:**
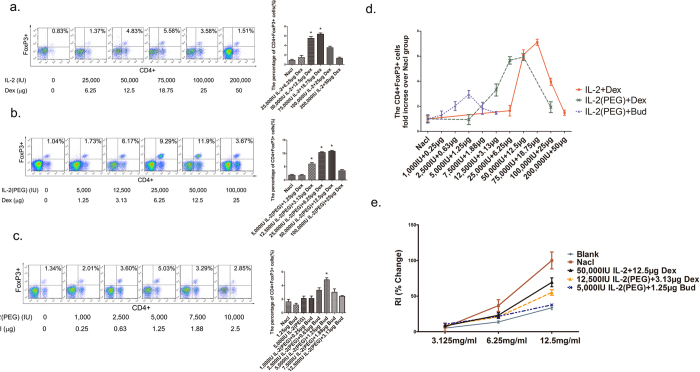
Treg cells and lung resistance analysis after administration of various drug combinations between glucocorticoids and IL-2. Female BALB/c mice were immunized with OVA i.p on days 1 and 8, followed by intranasal (i.n) 2% OVA challenges on days 9–14. Drugs were administrated intratracheally on days 12–14. On day 15, mice were sacrificed and analyzed. (**a–c**) Detection of CD4+FoxP3+ Treg cells after 3 days of treatment with different doses of IL-2 plus dexamethasone (Dex) (**a**), IL-2(PEG) plus Dex (**b**) and IL-2(PEG) plus budesonide (Bud) (**c**) in asthma model mice with a fixed ratio between two drugs (40,000 IU IL-2: 1 μg glucocorticoid). The upregulation of Treg cells was dose-dependent and different dosage forms could upregulate Treg cells and alleviate asthma in various doses. (**d**) Synthetic analysis of the upregulation of Treg cells in three dosage forms. (**e**) AHR analysis between treated and untreated mice. Results represent the changes in lung resistance (Rl) as a measure of AHR. *p < 0.05. (**a–c**) Data are presented as means ± SEM (n = 8 per group and data point). Treated group versus untreated group by Student’s *t* test. (**e**) Data are presented as means ± SEM (n ≥ 4 per group and data point); here representative results from 1 of 2 experiments are shown. Treated group versus blank group by Student’s *t* test. Blank group, health control mice. Nacl group, asthma model mice treated with normal saline.

Our previous study suggested that the upregulation of Treg cells was dose-dependent and, therefore, determined the optimal dose of IL-2 combined with dexamethasone^11^. Based on a fixed ratio (4,000 IU IL-2: 1 μg dexamethasone), we explored the upregulation of Treg cells in BALF from an asthma mouse model at different doses. The proportion of Treg cells among lymphocytes rose with increasing doses, and a 12.5 μg of dexamethasone with a corresponding dose of 50,000 IU IL-2 was effective at upregulating Treg cells, which is a relatively low dose (Fig. 2a). However, the required dose could be too high in clinical use.

Conjugation of macromolecules to polyethylene glycol (PEG) can increase the retention of drugs in the body by protecting against enzymatic digestion, slowing filtration by the kidneys and reducing the generation of neutralizing antibodies, which makes it a potential strategy to improve therapeutic effects^14^. Thus, we replaced the traditional IL-2 with a new one modified by PEG (IL-2(PEG)) and it markedly lowered the effective dose to 12,500 IU IL-2(PEG) plus 3.13 μg dexamethasone (Fig. 2b). However, dexamethasone is not a regular inhaled corticosteroid, so we replaced dexamethasone with budesonide, and the effective dose was further reduced to 5,000 IU IL-2(PEG) plus 1 μg budesonide (Fig. 2c), which is a relatively low and ideal dose for trial of the potential therapy for use in humans. Synthetic analysis indicated that the change of dosage form of glucocorticoid or IL-2 could both help lower the effective dose, and the range of expansion for Treg cells mainly depended on the doses of IL-2 (Fig. 2d). We also measured the reduction of airway hyperresponsiveness (AHR) in asthma mice model treated with effective doses of different dosage forms. Although the upregulation of Treg cells was different, all the dosage forms successfully reduced the AHR, and IL-2(PEG) plus budesonide was most effective at the lower dose, which we took as the optimal dosage form (Fig. 2e).

### Different ratios of IL-2(PEG): budesonide can differentially upregulate Treg cells and combined administration exhibits a broad effective extent

**Figure 3:**
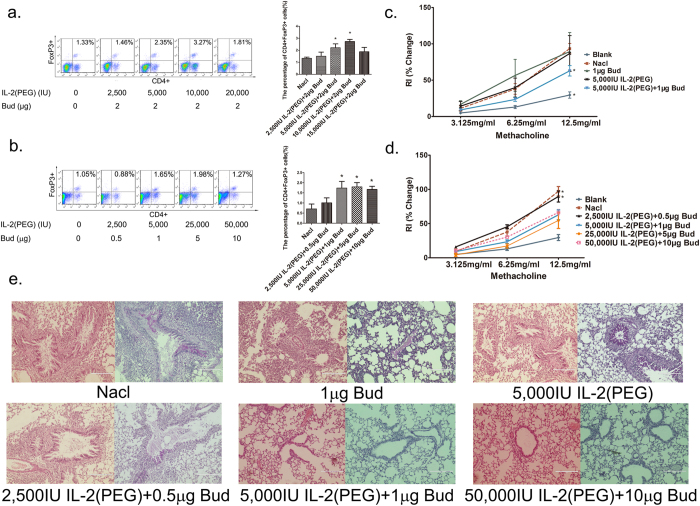
Treg cell, lung resistance and lung tissue analysis after intervention with various ratios or doses of IL-2(PEG) and budesonide. Female BALB/c mice were immunized with OVA i.p on days 1 and 8, followed by intranasal (i.n) 2% OVA challenges on days 9–14. Drugs were administrated intratracheally on days 12–14. On day 15, mice were sacrificed and analyzed. (**a**) Treg cell composition was analyzed by flow cytometry after intratracheal administration of various ratios of IL-2(PEG) and budesonide(Bud) for 3 days in asthma model mice. It showed that a ratio of 5,000 IU IL-2(PEG):1 μg Bud was optimal. (**b**) Treg cell analysis after intratracheal administration of different doses of IL-2(PEG) plus Bud combined in a fixed ratio of 5,000 IU IL-2(PEG):1 μg Bud for 3 days in asthma model mice. (**c–e**) AHR measurement and images of lung sections (scale bars, 200 μm) in asthma model mice treated with different drugs. Results represent the changes in lung resistance (Rl) as a measure of AHR. *p < 0.05. (**a,b**) Data are presented as means ± SEM (n = 8 per group and data point). Treated group versus untreated group by Student’s *t* test. (**c**) Data are presented as means ± SEM (n ≥ 4 per group and data point); here representative results from 1 of 2 experiments are shown. Other group versus Nacl group by Student’s *t* test. (**d**) Data are presented as means ± SEM (n ≥ 4 per group and data point); here representative results from 1 of 2 experiments are shown. Treated group versus blank group by Student’s *t* test. (**e**) Left, H&E staining; right, PAS staining. Blank group, health control mice. Nacl group, asthma model mice treated with normal saline.

We had determined the combination of IL-2(PEG) and budesonide as the best dosage form, but with different working characteristics between different dosage forms and administration routes, the best ratio of two drugs remained to be illuminated. Using a fixed 2-μg dose of budesonide per asthma model mouse, we were able to show that a corresponding dose of 10,000 IU IL-2(PEG) per mouse was optimal for Treg cell upregulation. That is to say, a ratio of 5,000 IU IL-2(PEG): 1 μg budesonide was optimal (Fig. 3a). Next, we evaluated the effective doses for such a ratio of two drugs by detection of Treg cells. Compared with the ratio of 4,000 IU IL-2(PEG): 1 μg budesonide we used before, the new ratio exhibited a broader effective extent, ranging from 5,000 IU IL-2(PEG) plus 1 μg budesonide to at least 50,000 IU IL-2(PEG) plus 10 μg budesonide (Fig. 3b). Then we analyzed the therapeutic effect of IL-2(PEG) combined with budesonide by measurement of AHR. Compared with treatment with IL-2(PEG) or budesonide alone, intratracheal treatment with a combination of 5,000 IU IL-2(PEG):1 μg budesonide markedly reduced AHR of asthma model mice (Fig. 3c). We also measured the AHR of asthma model mice treated with a high dose (50,000 IU IL-2(PEG):10 μg budesonide), a medium dose (25,000 IU IL-2(PEG):5 μg budesonide), a low dose (5,000 IU IL-2(PEG):1 μg budesonide) of drugs and a dose of 2,500 IU IL-2(PEG) plus 0.5 μg budesonide which failed to upregulated Treg cells in BALF. The results showed that 2,500 IU IL-2(PEG) plus 0.5 μg budesonide failed to ameliorate lung resistance, which met the results of Treg cells, suggesting that the expanded Treg cells alleviates allergenic airway disease. And all other three doses successfully ameliorated lung resistance, abrogated subsequent airway and tissue inflammation and reduced airway mucus plugging (Fig. 3d,e).

### IL-2(PEG) combined with budesonide can achieve the same curative effect as regular therapy and the effect can last for at least 6 weeks

**Figure 4:**
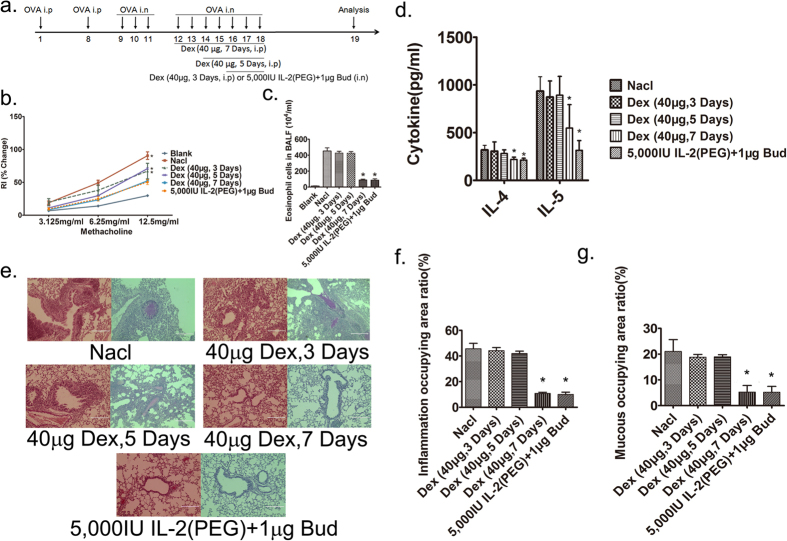
Manifestations of allergic airway disease after administration of different drugs. IL-2(PEG) combined with budesonide can achieve the same curative effect as regular therapy of systemic use of dexamethasone. (**a**) Timeline of drug intervention and analysis. Female BALB/c mice were immunized with OVA i.p on days 1 and 8, followed by intranasal (i.n) 2% OVA challenges on days 9–14. For i.n group, 5,000 IU IL-2(PEG) plus 1 μg budesonide were administrated intratracheally on days 16–18. For i.p groups, 40 μg dexamethasone was injected intraperitoneally on days 12–18, 14–18 or 16–18. On day 19, mice were sacrificed and analyzed. (**b–g**) To prove the efficacy of the combination of IL-2(PEG) plus budesonide compared with traditional treatment, we measured AHR, eosinophil counts and Th2 cytokines IL-4 and IL-5 in BALF and images of lung sections (scale bars, 200 μm) in asthma model mice treated with 40 μg dexamethasone (Dex) intraperitoneally for 3, 5 or 7 days or treated with 5,000 IU IL-2(PEG) plus 1 μg budesonide (Bud) for 3 days. Results represent the changes in lung resistance (Rl) as a measure of AHR. *p < 0.05. (**b–d**,**f**,**g**) Data are presented as means ± SEM (n ≥ 4 per group and data point); here representative results from 1 of 2 experiments are shown. Treated group versus blank group (**c**) or Nacl group (**d**) by Student’s *t* test. (**d**) Left, H&E staining; right, PAS staining. *i.n*., intranasal; *i.p*., intraperitoneal. Blank group, health control mice. Nacl group, asthma model mice treated with normal saline.

Injection of dexamethasone is an effective and accepted method to alleviate asthma symptoms, especially for severe cases. In this study, we discovered that it took at least 7 days before a serial injection of dexamethasone (40 μg per mouse) successfully reduced the AHR. However, a low dose of IL-2 (PEG) plus budesonide (5,000 IU: 1 μg) could ameliorate lung resistance to the same level within 3 days via intratracheal administration (Fig. 4b).We also evaluated eosinophil counts and Th2 cytokines IL-4 and IL-5 in murine BALF and found them significantly decreased after 3-days intratracheal use of 5,000 IU IL-2(PEG) plus 1 μg budesonide or 7-days injection of 40 μg dexamethasone (Fig. 4c,d). Additionally, airway inflammation and mucus production also decreased in these two groups (Fig. 4e–g). However, a 3-days or 5-days injection of 40 μg dexamethasone did not have a similar response. This finding suggests that short-term intratracheal use of IL-2(PEG) plus budesonide at a low dose can achieve the same therapeutic efficacy as conventional treatment.

**Figure 5:**
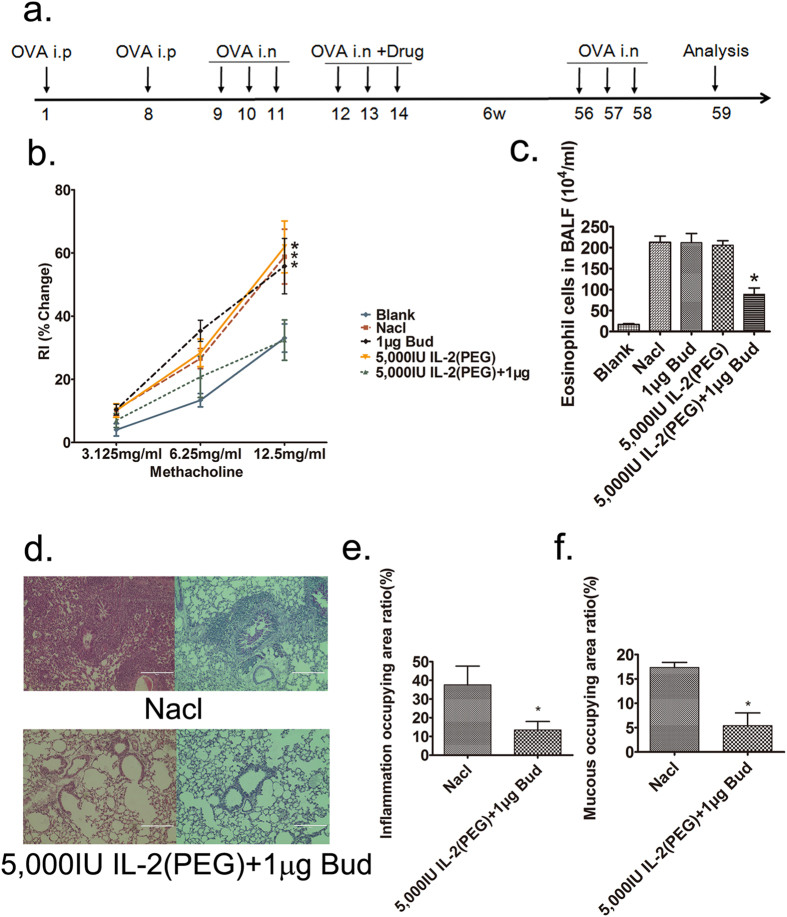
Manifestations of allergic airway disease 6 weeks after intratracheal use of IL-2(PEG) plus budesonide. The therapeutic effect of intratracheal use of IL-2(PEG) plus budesonide could last for at least 6 weeks. (**a**) Timeline of drug intervention and analysis. Female BALB/c mice were immunized with OVA i.p on days 1 and 8, followed by intranasal (i.n) 2% OVA challenges on days 9–14. Drugs were administrated intratracheally on days 12–14. On days 56–58, mice were challenged with 2% OVA for three days again. And on day 59, mice were sacrificed and analyzed. (**b–f**) To prove that the amelioration of airway inflammation can last a long time, we measured AHR, eosinophil cells counts and Th2 cytokines IL-4 and IL-5 in BALF and images of lung sections (scale bars, 200 μm) in asthma model mice 6 weeks after intratracheal administration with 5,000 IU IL-2(PEG) plus 1 μg Bud for 3 days (n ≥ 4 per group). Results represent the changes in lung resistance (Rl) as a measure of AHR. *p < 0.05. (**b,c,e,f**) Data are presented as means ± SEM (n ≥ 4 per group and data point); here representative results from 1 of 2 experiments are shown. Treated group versus blank group (**b**) or Nacl group (**d**) by Student’s *t* test. (**d**) Left, H&E staining; right, PAS staining. *i.n*., intranasal; *i.p*., intraperitoneal. Blank group, health control mice. Nacl group, asthma model mice treated with normal saline.

BALB/c asthma model mice were administrated 5,000 IU of IL-2(PEG) plus 1 μg of budesonide or saline intratracheally for 3 days. After 6 weeks, all the mice were challenged with 2% OVA for 3 days before being anesthetized, tracheostomized and mechanically ventilated and lung resistance was measured. We found that the treated mice still showed a lower AHR, decreased eosinophi counts, reduced broncho-vascular inflammation and fewer mucus-producing goblet cells compared with the saline group (Fig. 5), even the upregulation of Treg cells failed to be detected after such a long term (see Supplementary Fig. S1). However, there were no statistical differences between pathological manifestations in other intervention groups.

### Mechanism of alleviating asthma by combination of IL-2 and glucocorticoid

**Figure 6:**
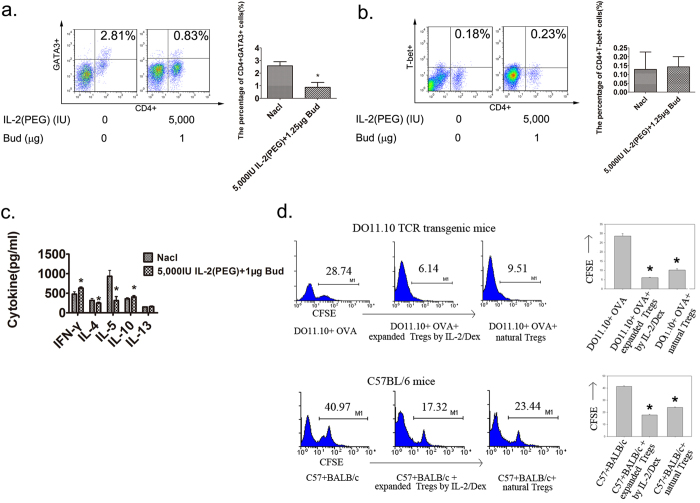
Detection of Th2 cell and cytokines in BALF. Measurements of expanded Treg cells for antigen-specificity. The mechanism of combined use of glucocorticoid and IL-2 in alleviating asthma rested on expanding antigen-nonspecific Treg cells, with a decrease in T helper 2 (Th2) cells and Th2-associated cytokines in the airway. Female BALB/c mice were immunized with OVA i.p on days 1 and 8, followed by intranasal (i.n) 2% OVA challenges on days 9–14. 5,000 IU IL-2 plus 1 μg budesonide (Bud) were administrated intratracheally on days 12–14. On day 15, mice were sacrificed and analyzed by flow cytometry and ELISA. And Treg cells were purified on day 15. (**a,b**) Detection of CD4+GATA3+ Th2 cell and CD4+T-bet+ Th1 composition among lymphocytes in BALF by flow cytometry. (**c**) Measurements of cytokines (IL-4, IL5, IL-10, IL-13 and IFN-γ) by ELISA in BALF. (**d**) Expanded Treg or Treg cells were purified from BALF of asthma model mice after treatment with IL-2 plus dexmethasone (Dex) for 3 days or the spleens of healthy BALB/c mice to be measured for antigen-specificity in OVA-specific proliferation assays and mixed lymphocyte reactions. *p < 0.05. (**a,b,c**) Data are presented as means ± SEM (n = 6 per group and data point) from 2 independent experiments. Treated group versus untreated group by Student’s *t* test. (**c**) Data are presented as means ± SEM (n = 3 per group and data point); here representative results from 1 of 2 experiments are shown. Treg cells addition group versus non-Treg cells group by Student’s *t* test.

After a 3-day administration of IL-2(PEG) and budesonide in asthma BALB/c mice, the BALF samples were obtained. The proportion of T helper 2 (Th2) cells among the lymphocytes were analyzed by flow cytometry and the cytokines (IFN-γ, IL-4, IL-10 and IL-13) in BALF were measured by ELISA. We found that, as the proportion of Treg cells increased, which of Th2 cells decreased markedly (Fig. 6a). In addition, we failed to observe an upregulation of Th1 cells in BALF (Fig. 6b). Accordingly, cytokines associated with suppressed function in asthma (IFN-γ and IL-10) which could be secreted by Treg cells increased in the treated compared with the untreated group, whereas cytokines associated with attack and progression of asthma (IL-4 and IL-5) decreased. However, as an important cytokine that promotes asthma, IL-13 failed to exhibit any difference between the two groups (Fig. 6c). Furthermore, after delivering of glucocorticoid and IL-2, the expression of FoxO3a, which could be induced by use of glucocorticoid^15^, was higher than control group. At the same time, IL-2 caused more phosphorylation of Stat5 as what has been reported before^16^, with lower unphosphorylated Stat5 accordingly (see Supplementary Fig. S2).

To determine whether Treg cells expanded by drugs are antigen-specific, CD4+CD25+ Treg cells were purified from the BALF of asthma model mice after administration of IL-2 and dexamethasone for 3 days. Lymphocytes isolated from DO11.10 OVA-transgenic mice were stimulated by OVA, but the proliferation was inhibited by the purified Treg cells from BALF as well as natural (nTreg) cells isolated from spleens. Furthermore, these Treg cells also played an important role in inhibiting lymphocyte proliferation in mixed lymphocytes reactions as nTreg cells, which suggested that the Treg cells expanded by IL-2/dexamethasone were not antigen-specific (Fig. 6d).

### Intratracheal use of IL-2(PEG) combined with budesonide has profound effects with fewer side effects

**Figure 7:**
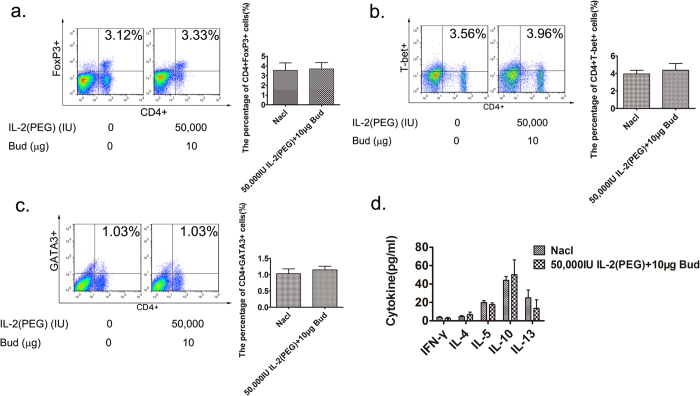
Detection of Th2 and Treg cells and cytokines after drug intervention. The combined use of glucocorticoid and IL-2 had no obvious impact on the whole body. Female BALB/c mice were immunized with OVA i.p on days 1 and 8, followed by intranasal (i.n) 2% OVA challenges on days 9–14. 50,000 IU IL-2 plus 10 μg budesonide (Bud) were administrated intratracheally on days 12–14. On day 15, mice were sacrificed and analyzed by flow Tcytometry or ELISA. (**a–c**) Detection of CD4+FoxP3+ Treg cells, CD4+T-bet+ Th1 cells and CD4+GATA3+ Th2 cells in spleens after 3 days use of 50,000 IU IL-2(PEG) combined with 10 μg budesonide (Bud) by flow cytometry. (**d**) Measurement of cytokines by ELISA in serum after 3 days use of 50,000 IU IL-2(PEG) combined with 10 μg Bud. Data are presented as means ± SEM (n ≥ 6 per group and data point) from 2 independent experiments. p > 0.5. Treated group versus untreated group by Student’s *t* test.

In our previous study, intraperitoneal use of IL-2 plus dexamethasone in the short term was effective at alleviating airway inflammation. However, it changed Treg/Th2 in spleen, which might interfere with anti-tumor or anti-infection immune responses, resulting in a series of side effects^11^. In this study, we analyzed the proportion of Treg, Th1 and Th2 cells in spleens and associated cytokines in peripheral blood samples after 3 days of intratracheal treatment with high-dose IL-2(PEG) and budesonide (50,000 IU IL-2(PEG): 10 μg budesonide). We found that, even with such a high-dose inhalation of drugs, local administration did not significantly change the proportion of Th1,Th2 and Treg cells or the levels of associated cytokines for whole body (Fig. 7). However, a 3 days-injection of 40 μg dexamethasone could significantly decreased the propotion of Th1, Th2 and Treg cells in spleen, which may result in an immune imbalance (see Supplementary Fig. S3).

## Discussion

The increasing incidence of asthma has become a worldwide problem. Immunotherapy is the only available cure for asthma, but it has limitations and side effects^17^,^18^,^19^. Treg cells play a very important role in maintaining immune homeostasis and the defective or overwhelmed suppression of Treg cells is responsible for asthma attacks^20^. Many investigators have managed to control airway inflammation by injection of Treg cells, but the isolation of Treg cells *in vitro* has restricted application in the clinic^6^,^7^. Other pharmacological manipulations have attempted to upregulate Treg cells to prevent the disease^8^, but there is no evidence to support whether such a method works in pathological conditions.

In this study, we found that a combination of glucocorticoid and IL-2 administered intratracheally in the short term was consistently effective for the upregulation of Treg cells in the airways and for amelioration of airway inflammation and AHR. Different kinds of glucocorticoid are often clinically used for the treatment of asthma, but this treatment only relieves symptoms and does not reverse the progression or cure the disease, especially in severe cases^21^. Moreover, the therapeutic effect of glucocorticoids depends on sustained use, and even inhaled use can cause significant systemic activity and leads to multiple side effects^22^. Although short-term use of glucocorticoids succeeds in upregulating Treg cells^9^, frequent use might have an opposite effect of inducing a reduction of Treg cells in the lungs and lymphoid organs of allergen-challenged mice, or even intranasal application of glucocorticoids could limit Treg cells responses^23^,^24^. On the other hand, the use of IL-2 alone promotes the progression of asthma rather than ameliorating it^8^. However, the combined use of IL-2 and glucocorticoid intraperitoneally in a short term has proven to be useful in suppressing EAE and asthma, and the effect can last for a long time^10^,^11^. In this study, we found that a combination of glucocorticoid and IL-2 administered intratracheally in the short term was consistently effective for the upregulation of Treg cells in the airways and for amelioration of airway inflammation and AHR, and the alleviation of allergic airway diseases had disappeared when the upregulation of Treg cells had disappeared, suggesting the Treg cells were responsible for the observed effects. Compared with IL-2 and dexamethasone, IL-2(PEG) plus budesonide is the optimal dosage form, as it exhitites a stronger effect in terms of lowering AHR at a lower dose. Even though IL-2 (PEG) plus budesonide induced lower propotion of Treg cells in BALF compared to IL-2 or IL-2 (PEG) plus dexamethasone, the range of upregulation was not specific for disease remission. A possible explanation for this is that the upregulation level of Treg cells responses to the dose of IL-2 rather than glucocorticoid. The higher the dose of IL-2 administrated, the higher the upregulation of Treg cells. Both the PEG-modified IL-2 and budesonide could help lower the effective dose, which resulted in a lower upregulation of Treg cells instead. Furthermore, except for the inflammatory site, other parts of body were not significantly influenced by such short-term and local use. Except for the inflammatory site, T cells and cytokines in spleen or serum were not obviously influenced by such short-term and local use. Even the Th1 cells^25^, which were shown to be more susceptible to steroid, survived from such treatment.

Additionally, the sustained suppression of inflammation that results from an upregulation of Treg cells can last for at least 6 weeks, even the upregulation was unable to be detected after such a long term. The reason may be the primary effects of Treg cells in the previous upregulation. That is, at the first upregulation, Treg cells has worked to suppress the immune response, inhibit the secretion of proinflammatory cytokines from Th2 cells and, reduce the recruitment of inflammatory cells like eosinophil or mast cells, and the down-regulation of Th2 cells resultes in a moderate pathological process of asthma. All above leads to a long-term effect. When the asthma model mice were challenged with antigen for the second time, the immune response was relatively weaker, making it possible to take combined use of IL-2 and glucocorticoid as a long-term and convenient methodology for asthma.

The expanded Treg cells in BLAF may consist of natural Treg cells and adaptive Treg cells. Theses expanded Tregs following the combination treatment inhibited both allergen-specific and non-specific immune responses, which means that such Treg cells could prevent asthma induced by other antigens, and the expanded cells were likely natural Treg cells. However, the ELISA results showed increased IL-10 in BALF, which was produced by adaptive Treg cells to mediate their inhibitory activites^26^. In our study, both natural Treg cells and adaptive Treg cells were activated in response to asthma, induced and protected by drug administration, and they worked together to alleviate asthma.

An explanation for the upregulation of Treg cells rests on the differential levels of response to glucocorticoids in the presence of IL-2 between CD4+CD25+ and CD4+CD25- cells. As a member of the Forkhead family, FoxO3a regulates genes that mediate potent pro-apoptotic signals following different stimuli^27^. Phosphorylated FoxO3a is largely excluded from the nucleus and anchored in the cytoplasm while unphosphorylated FoxO3a, the activated form, almost entirely translocates into the nucleus and induces the transcription of genes encoding pro-apoptotic and anti-proliferative proteins^28^,^29^,^30^. By promoting the expression and inhibiting the phosphorylation of FoxO3a, glucocorticoid induces apoptosis in lymphocytes^31^. Compared with CD4+CD25- T cells, CD4+CD25+ T cells are more resistant to glucocorticoid in the presence of IL-2 and the explanation may rest on their different response to the concentration of IL-2. IL-2 acts on either the high-affinity trimeric or the low-affinity dimeric IL-2R^32^. The dimeric IL-2R consists of CD122 (also known as IL-2Rβ) and the common cytokine receptor γ chain (γ_c_; also known as CD132), whose affinity for IL-2 is weak at about 10^−9 ^mol/L. However, trimeric IL-2R includes an additional CD25 (also known as IL-2Rα), which does not participate in signaling but increases the affinity of IL-2R for the ligand by 10–100-fold, suggesting that a lower concentration of IL-2 (K_d_≈10^−11^) can stimulate IL-2R^33^,^34^,^35^. The stimulation of IL-2R activates Akt and SGK, which can phosphorylate FoxO3a into an inactivated form, preventing the cells from apoptosis^11^. Expression of CD25 is almost undetectable on CD4+CD25- naïve T cells, but at a high level on CD4+CD25+ Treg or effector T cells, making them sensitive to exogenously administered IL-2, which protected them from the glucocorticoid-induced apoptosis. In the circumstance of glucocorticoid and a low concentration of IL-2, we can observe the induction of apoptosis by glucocorticoid of CD4+CD25- T cells, while CD4+CD25+ T cell were protected by IL-2, resulting in upregulation of CD4+CD25+ Treg cells and inhibition of Th2 differentiation. Doganci *et al*.^36^ found that i.n. administration of Abs against the IL-2Rβ ameliorated both inflammation and airway hyperresponsiveness in experimental allergic asthma, which could be explained by the different distribution of CD25 and CD122 between various T cells, too. CD4+CD25- naïve T cells were inhibited by Abs against the IL-2Rβ, while CD4+CD25+ Treg cells were still sustained by IL-2. Additionally, IL-2 is important for the survival and homeostasis of Treg cells^37^, which contributes to the upregulation of Treg cells as well. As the concentration of IL-2 increases, the selective activation of IL-2R disappears, and CD4+CD25- could also be protected from apoptosis by IL-2R, which resulted in lower upregulation of Treg cells in this study, as what we have reported before^11^. Furthermore, a high concentration of IL-2 even program T cells for apoptosis^38^.

The combined use of IL-2 and glucocorticoid significantly reduced the Th2 cytokines IL-4 and IL-5 in BALF with a down-regulation of Th2 cells, while we failed to observe a decrease of another important Th2 cytokine IL-13. In the pathogenesis of patients with atopic asthma, IL-13 could be secreted by active Th2 cells^39^, mast cell^40^, NK T cells^41^, NK cells^42^ and so on. We hypothesize that as a responder to IL-2^43^, NK cell could be activated in the circumstance of IL-2 and secreted several associated cytokines, including IL-13. It might be the reason why IL-13 showed no changes. Because cell component in BALF is too complex to be detected in details, more experiments may be done in the future to further elucidate the mechanism.

In this study, we creatively used a PEG-modified IL-2 instead of traditional recombinant human IL-2 to enhance the curative effect at a lower dose. Moreover, intratracheal rather than systemic administration not only helped further lower the therapeutic dose but also made it practical for clinical application, characterized by hypotoxicity and less invasiveness. We believe that such an effective therapy could greatly benefit patients with allergic airway disease in the future.

## Methods

### Animals

Female BALB/c, OVA-specific DO11.10 transgenic mice and male C57BL/6 mice, 6–8 weeks old, were purchased from Shanghai Laboratory Animal Center and raised in the animal department of the Institute of Biochemistry and Cell Biology, Chinese Academy of Sciences, Shanghai. Mice were maintained in pathogen-free conditions and fed with standard laboratory food and water ad libitum. All the animal experiments were approved by the Institutional Animal Care and Use Committee of the Institute of Biochemistry and Cell Biology, Chinese Academy of Sciences, Shanghai, and performed in accordance with institutional and state guidelines (IACUC:2013-084).

### Preparation of PEG-modified IL-2

After ultrafiltration, recombinant human IL-2 (Xiamen Amoytop Biotech, Xiamen, China) was dissolved in sodium acetate buffer solution. IL-2 and a kind of mPEG-propionaldehyde, M-AlD-20 K were mixed under a mass mixing ratio of 1:5. After 12 h of modification reaction, the PEG-modified IL-2 (IL-2 (PEG)) was purified by chromatography (see Supplementary Fig. S4).

### Immunization and intervention

This protocol was followed as previously described^11^. Briefly, mice were immunized intraperitoneally with OVA (100 μg; Sigma-Aldrich, St Louis, MO, USA) and aluminum hydroxide (2 mg; Pierce, Rockford, IL, USA) in sterile saline on day 1 and 8. On day 9 to 14 after the initial sensitization, mice were challenged intranasally with 20 μg of 2% OVA in sterile saline. Recombinant human IL-2 (Xiamen Amoytop Biotech) and dexamethasone (Cisen pharma, Shandong, China) or budesonide (Astrazeneca, NorthRyde, Australia) were intratracheally sprayed from the tip of the micro-sprayer (Model IA-1C, PennCentury, Wyndmoor, PA) attached to a syringe inserting into the trachea under visual guidance following anesthesia.

### Collection of bronchoalveolar lavage fluid (BALF) and flow cytometry

Mice were killed 24 h after the last drug administration and the trachea was cannulated and airspaces were lavaged with an initial 300 μl of sterile PBS, followed by two 300-μl PBS washes. The cells in the BALF were collected and stained with FITC-conjugated CD4, APC-conjugated FoxP3, Per-CP-conjugated GATA3 and PE-conjugated T-bet as described in the protocol, using the Mouse Regulatory T Cell Staining Kit (eBioscience, San Diego, Calif). The expression of surface markers and intracellular proteins were analyzed by a FACSCalibur flow cytometer (Becton Dickinson, USA) and a FlowJo software (Treestar, USA).

### ELISA

The peripheral blood samples were stored at 37 °C for 1 h before centrifugation (8,000 rpm, 5 min), and the serum was collected in new tubes. The BALF samples were also centrifuged and the supernatants were collected in new tubes. The cytokines were measured by using IFN-γ, IL-4, IL-10 and IL-13 ELISA kits (R&D Corp, Minneapolis, Minn).

### Lung histopathology and computer-based quantification of inflammation and mucus production

Lung tissues were fixed in 10% formalin and embedded in paraffin for sectioning. Hematoxylin and eosin (H&E; Merck & Co, Inc, Whitehouse Station, NJ) staning or Alcian blue/periodic acid–Schiff (PAS; Sigma-Aldrich) staining were used to analyze airway inflammation and pathological processes in the mice. For quantification and objective evaluation of the degree of histological inflammation and mucus production, lung sections were scanned with a digital camera (AMG EVOS system; 5 shots per lung) and analyzed with Image J software. The levels of inflammation were evaluated by two individual investigators.

### Measurement of airway hyperreactivity

Mice were anesthetized with pentobarbital sodium, tracheostomized and mechanically ventilated as described previously^44^. Lung resistance change was measured using invasive BUXCO (BUXCO Electronics, Troy, New York) in response to increasing doses (3.125 mg/ml, 6.25 mg/ml and 12.5 mg/ml) of aerosolized methacholine (Sigma-Aldrich).

### Purification of cells, OVA-specific proliferation assay and mixed lymphocyte reaction

After 3 days of drug administration of IL-2 combined with glucocorticoid in asthma model mice, CD4+CD25+ T cells were isolated from BALF using the CD4+CD25+ murine regulatory T Cell Isolation Kit (Miltenyi Biotech, Bergisch Gladbach, Germany). At the same time, natural Treg (nTreg) cells were isolated from the spleens of healthy BALB/c and DO10.11 mice. The purity of CD4+CD25+ T cells was greater than 90%, and that of CD4+CD25+ T cells was greater than 95%.

Cells were cultured in 96-well round-bottom plates (Costar, New York, NY, USA) in a total volume of 200 μl RPMI 1640 medium with 10% FBS, 100 U/ml penicillin, and 100 μl/ml streptomycin (GIBCO,Gaithersburg, MD, USA). OVA-specific proliferation assay and mixed lymphocyte reaction were conducted as previously described^45^,^46^. For OVA-specific proliferation assay, about 2 × 10^5^ splenocytes were loaded with 0.5 μM OVA. For mixed lymphocyte reaction assay, fixed numbers of responder cells (separated from BALB/c mouse splenocytes) and irradiated allogeneic stimulator cells (splenocytes of C57BL/6 mice) (10^5 ^cells, respectively) were mixed. A fixed number (1:5 to reactive cells) of CD4+CD25+ Treg cells (separated from spleens of heathy BALB/c mice or the BALF of treated asthma model mice) were added to the culture. After 3 days of co-culture, cell pellets were collected, washed and stained with CD4-allophycocyanin for FACS analysis of CFSE fluorescence.

### Statistical analysis

Data sets were expressed as mean ± SEM, and analyzed by 2-tailed Student *t* test or two-way ANOVA analysis. Differences were considered significant at p < 0.05.

## Additional Information

**How to cite this article**: Wu, K. *et al*. Short-term intratracheal use of PEG-modified IL-2 and glucocorticoid persistently alleviates asthma in a mouse model. *Sci. Rep.* **6**, 31562; doi: 10.1038/srep31562 (2016).

## Supplementary Material

Supplementary Information
