# Supplementary material for: Short-term intratracheal use of PEG-modified IL-2 and glucocorticoid persistently alleviates asthma in a mouse model
Source: Sci Rep. 2016 Aug 16;6:31562. doi: 10.1038/srep31562 (PMC4985708; doi:10.1038/srep31562)
Supplement: Supplementary Information [file srep31562-s1.pdf]

Short-term intratracheal use of PEG-modified IL-2 and glucocorticoid persistently alleviates asthma in a mouse model

Kefei Wu[1*], Jiexian Ma[1*], Weiya Bai[2], Xiaoxian Cui[2], Tao Han[1], Shiyuan Wang[3], Youhua Xie[2], Yanhui Xie[1]

[1]*Department of Hematology, Huadong Hospital, Fudan University, Shanghai 200040, People's Republic of China,* [2]*Key laboratory of medical molecular virology, Institutes of biomedical sciences and institute of medical microbiology, Fudan University, Shanghai 200032, People's Republic of China,* [3]*Xiamen Amoytop Biotech Co., Ltd, Xiamen 360000, People's Republic of China*

Correspondence: Yanhui Xie

E-mail: xyh@medmail.com.cn

Tel: +86 13321957387

Address: No.221 West Yan'an Road, Shanghai, China

* These authors contribute equally to this work.

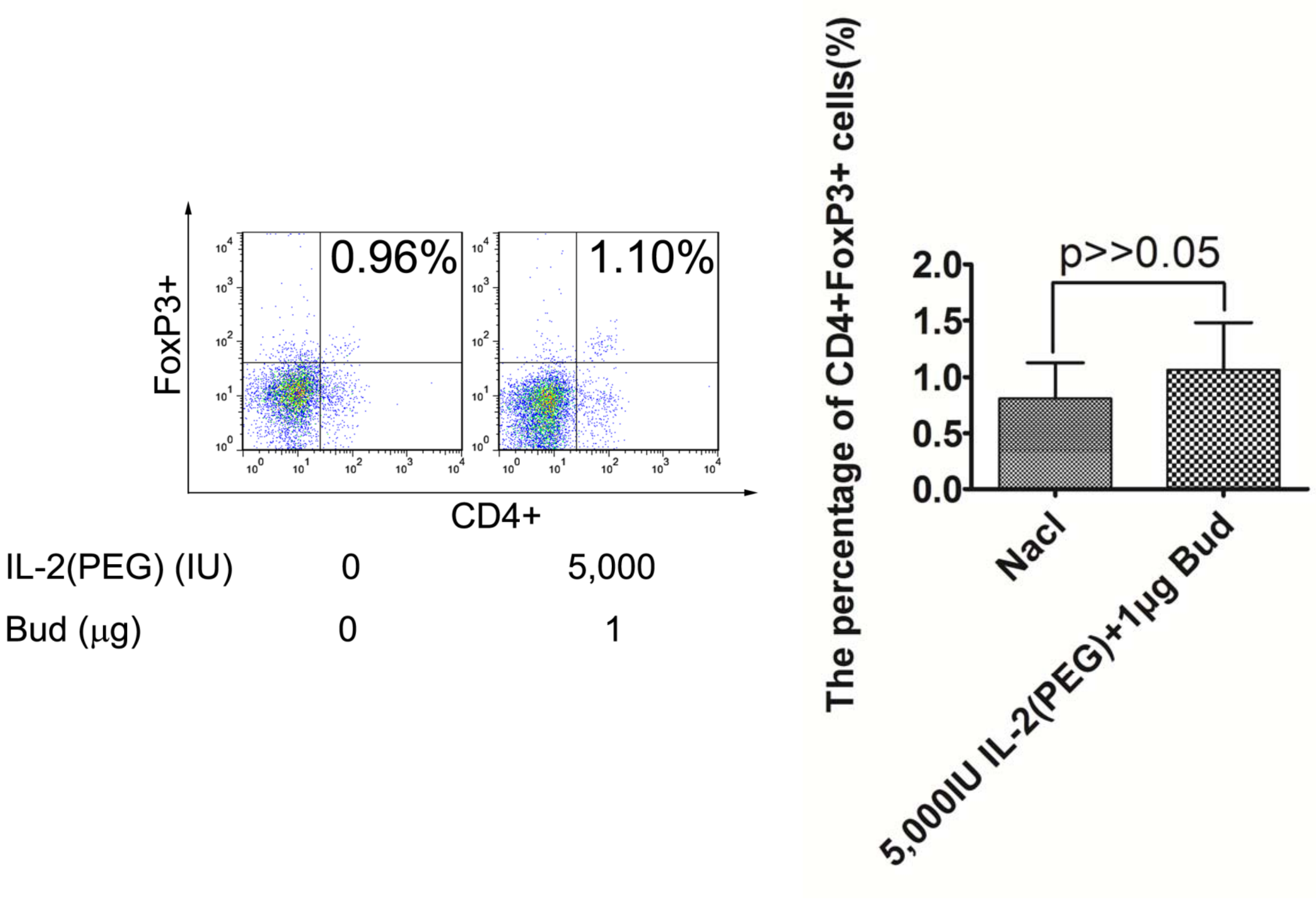

Supplementary Fig. S1. Detection of Treg cells in BALF 6 weeks after intratracheal use of IL-2(PEG) plus budesonide. Upregulation of Treg cells failed to be detected after 6 weeks. Data are presented as means ± SEM (n=5 per group and data point).

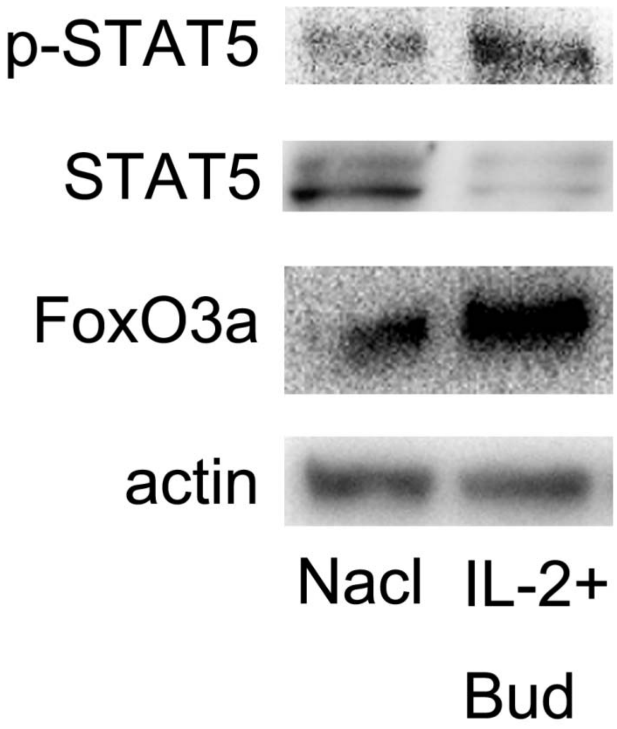

Supplementary Fig. S2. Expressions of p-Stat5, Stat5 and FoxO3a in the cells collected from BALF in the presence of the indicated treatment. Higher expression of FoxO3a and more phosphorylation of Stat5 could be detected. Phospho-Stat5, Stat5 and FoxO3a were analyzed by immunoblotting. Similar results were obtained in 2 independent experiments.

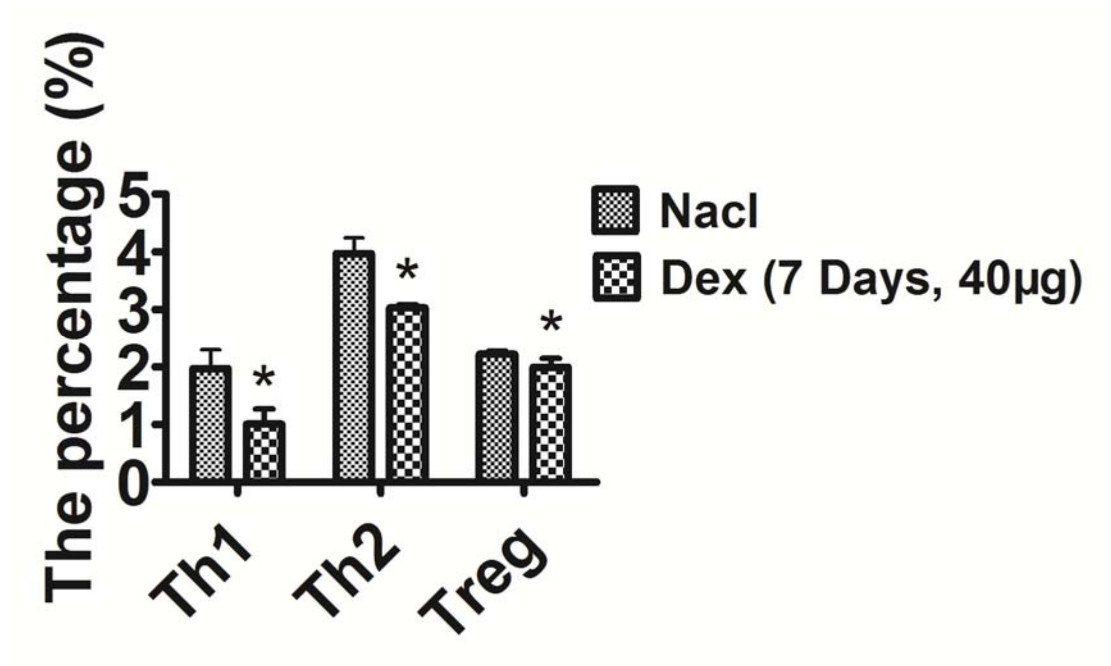

Supplementary Fig. S3. Detection of Th1, Th2 and Treg cells in spleen after intraperitoneal use of 40μg dexamethasone for 7 days. Down-regulation of Th1, Th2 and Treg cells could be detected. Data are presented as means ± SEM (n=5 per group and data point).

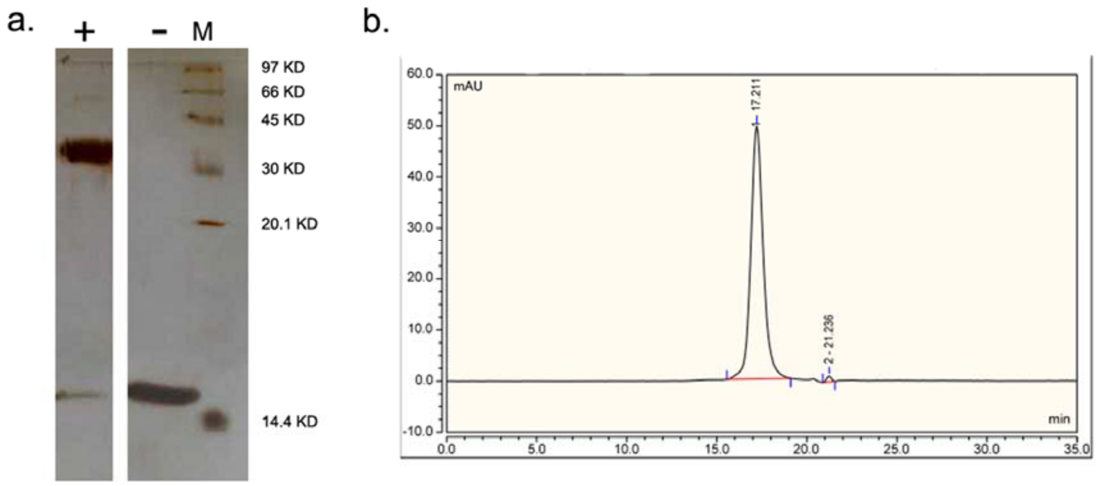

Supplementary Fig. S4. Production of PEG-modified IL-2. (a) Recombinant human IL-2 modified before (-) or after (+) modification with PEG monitored by SDS-PAGE followed with silver staining. (b) PEG-modified IL-2 after purification monitored by HP-SEC. M, marker.
